# Badges for sharing data and code at *Biostatistics: *an observational study

**DOI:** 10.12688/f1000research.13477.2

**Published:** 2018-03-07

**Authors:** Anisa Rowhani-Farid, Adrian G. Barnett

**Affiliations:** 1 Institute of Health and Biomedical Innovation, Queensland University of Technology, Brisbane, Queensland, 4001, Australia

**Keywords:** Reproducibility, incentives, rewards, data sharing, code sharing, meta-research

## Abstract

**Background**: The reproducibility policy at the journal *Biostatistics* rewards articles with badges for data and code sharing.  This study investigates the effect of badges at increasing reproducible research.

**Methods**:  The setting of this observational study is the *Biostatistics *and *Statistics in Medicine *(control journal) online research archives.  The data consisted of 240 randomly sampled articles from 2006 to 2013 (30 articles per year) per journal.  Data analyses included: plotting probability of data and code sharing by article submission date, and Bayesian logistic regression modelling.

**Results**:  The probability of data sharing was higher at *Biostatistics *than the control journal but the probability of code sharing was comparable for both journals.  The probability of data sharing increased by 3.9 times (95% credible interval: 1.5 to 8.44 times, p-value probability that sharing increased: 0.998) after badges were introduced at *Biostatistics*.  On an absolute scale, this difference was only a 7.6% increase in data sharing (95% CI: 2 to 15%, p-value: 0.998).  Badges did not have an impact on code sharing at the journal (mean increase: 1 time, 95% credible interval: 0.03 to 3.58 times, p-value probability that sharing increased: 0.378).  64% of articles at *Biostatistics* that provide data/code had broken links, and at *Statistics in Medicine*, 40%; assuming these links worked only slightly changed the effect of badges on data (mean increase: 6.7%, 95% CI: 0.0% to 17.0%, p-value: 0.974) and on code (mean increase: -2%, 95% CI: -10.0 to 7.0%, p-value: 0.286).

**Conclusions:**  The effect of badges at *Biostatistics* was a 7.6% increase in the data sharing rate, 5 times less than the effect of badges at *Psychological Science*.  Though badges at *Biostatistics* did not impact code sharing, and had a moderate effect on data sharing, badges are an interesting step that journals are taking to incentivise and promote reproducible research.

## Introduction

Historically, the replication of a scientific experiment has been the measure of its validity, however, not all experiments can be replicated in their totality ^1^. ‘Replicability’ is the ability of a researcher to duplicate the results of a prior study if the same procedures are followed but new data are collected ^2^. In 2009, Roger Peng mentioned in an editorial in *Biostatistics* that the minimum standard that could bridge the gap between replicability and nothing is “reproducible research” ^1^. ‘Reproducibility’ is the ability of a researcher to duplicate the results of a prior study using the same materials as were used by the original investigator ^2^. Reproducibility was defined by Peng in terms of sharing the data and computer code used to analyse the data and he described it as the “cornerstone of the scientific method” ^1^. In a perspective piece in 2011, Peng likened reproducibility to a spectrum, at one end being the gold standard of full replication, and at the other, publication only ^3^. Given the expectation that data will be accessible, researchers who refuse to share the evidentiary basis behind their conclusions, or the materials needed to reproduce published experiments, fail to maintain the standards of science ^4^. Although in some instances highly-sensitive data cannot be shared for legal or privacy reasons.

Scientific journals are critical to changing the culture of research. Many journals are introducing data sharing policies, but studies have shown that policies alone are not effective in promoting a culture of sharing and that scientists potentially need to be rewarded for good behaviour ^5^. Ioannidis *et al*. discuss changing the reward criteria to include ‘reproducible’ and ‘sharing’ using the PQRST criteria – productive, high-quality, reproducible, shareable, and translatable ^6^. A systematic review of incentives that motivated researchers to share their data in the health and medical research community, uncovered only one evidence-based incentive that increased data sharing at the journal *Psychological Science* from 1.5% pre-incentive (2012) to 39.4% post-incentive (2015) ^7, 8^. This incentive was an open data badge developed by the Center of Open Science (COS) and introduced at the journal in January 2014 ^8^.

Badges for reproducible research were not an innovative creation of COS however. The journal *Biostatistics* introduced badges, or what they called kitemarks (named after the UK kitemark system of establishing product safety), on 1 July 2009 as part of their policy to reward reproducible research ^1^. The policy was introduced by Roger Peng, the then Associate Editor for reproducibility (AER) ^1^. Sharing was not enforced, rather authors were encouraged to consider the reproducibility of their research ^1^. From here on, kitemarks will be referred to as badges, using common terminology.

The reproducibility policy at the journal instructed authors to indicate in their submission if they intend to submit supplementary materials that include data, code, or both ^1^. The policy rewarded articles with data available with the letter **D** on the front page of the published article PDF, articles with code available with a **C**, and articles with data and code available and which were tested for reproducibility by the AER an **R** for reproducibility ^1^. It is important to note that data refers to raw data and not simulated data, which are commonly used in statistics.

The policy change at *Biostatistics* provided an ideal opportunity to replicate the findings of the Kidwell *et al*. badge study by examining sharing rates at another journal that offered a reward or incentive for reproducible research ^8^. We note that Kidwell *et al*. examined data and material sharing only, as badges were not offered for code.

A survey conducted by *Nature* in 2016 indicates that the scientific community is in the midst of a reproducibility crisis ^9^. The current culture in science provides strong incentives for innovation and relatively weak incentives for certainty and reproducibility ^10^. Within the current ‘post-truth’ era there is much public scrutiny and suspicion around the validity of science. Such a debate, compounded by the reproducibility crisis, signals a time for a cultural shift in the scientific research process ^11^. The sharing of data, as well as the computer code used to analyse the data, should, where possible, be integral components of the research process, however data sharing rates have been as low as 0% ^12^. Of course, not all data can be shared due to legal and ethical constraints, but these are neither the only, nor main reasons behind low sharing rates ^13^. Scientists are still exploring the barriers towards sharing and a key concern is that researchers are not incentivised to share ^3^.

### Aim

Our aim is to investigate the effect of badges at increasing reproducible research, specifically, data and code sharing, at *Biostatistics*.

## Methods

### Participants

This is an observational study with two journals, intervention and control, using a pre-post study design, with 30 randomly selected papers per year from 2006 to 2013 for each journal. We chose *Statistics in Medicine* as the control journal as it did not have a badges or any type of reproducible research reward scheme during those years, but is in the same field of research with similar goals of publishing papers on statistical methods development in health and medicine. Additional control journals would have increased the representativeness of our study and increased the statistical power. However, no other similar journals from the field of biostatistics satisfied the inclusion criteria, as they all introduced a reproducibility policy before or between 2006 to 2013. Therefore, the study setting is the *Biostatistics* and *Statistics in Medicine* research archive. All the information required was publicly available online, as such participant consent was not required and an ethics exemption (exemption number: 1700001051) was granted by the Office of Research Ethics and Integrity at the Queensland University of Technology.

### Sample size calculation and power

A sample of only 19 papers per journal would have given us a 90% power to detect a difference in data sharing of 37.9%, based on the effect of badges from the Kidwell *et al*. study ^8^. This uses a two-sided 5% significance level. We felt this sample was unrealistically small, hence we instead based our sample size on the practical considerations of reading papers and examining their data and code sharing choices, given the time constraints of the first author’s (ARF) PhD. Thirty papers per year from 2006 to 2013 for two journals is a total sample of 480 papers, which is practically possible, and provides good coverage over the time of the policy change at *Biostatistics*.

### Data collection

For each year and journal, a random number generator was used to select the research articles (in Microsoft Excel 2016). Articles were included if they:

- Generated and analysed original data (article had data and code to share), or
- Conducted secondary analyses on a pre-existing dataset from another study (article had data and code to share), or
- Generated simulated data (article did not have data to share but had code to share)

Articles were excluded if:

- They were meta-analyses, meta-regressions, or systematic reviews, as these papers usually contain the data within the paper
- They were case series, opinion pieces or some other publication type without data or code

If an article was excluded then we sampled another article from the same year and journal to maintain the sample size. ARF read the research papers and extracted the details of the articles included in the study. Each article was screened using these search terms: “data”, “code”, “package”, “available”, “https”, “www”, “figshare”, and “github”. For the included articles, the following variables were documented: submission date, data sharing statement, data availability, hyperlink to dataset, code sharing statement, code availability, hyperlink to code, and badge allocation (for *Biostatistics* articles).

The second author (AGB) independently assessed data and code sharing for 20 randomly selected articles. There were minor discrepancies between the authors, which were resolved by discussion.

Using definitions from our previous work ^5^, each research article was categorised for data and code sharing as:

*Data sharing*

**available**: articles that had a functioning link to a publicly available dataset deposited at a third-party site or attached as supplementary material to the electronic version of the article

**potentially available**: articles that indicated that the dataset was potentially available upon request from the authors

**not available**: articles that did not indicate the availability of the dataset analysed in the article or where the link to the data was no longer working

**none to share**: articles that used simulated data and so did not have a raw dataset to share

*Code sharing*

**available**: articles that had a functioning link to publicly available code deposited at a third-party site, or attached as supplementary material to the electronic version of the article or available within the article itself

**potentially available**: articles that indicated that the code was potentially available upon request from the authors

**not available**: articles that did not indicate the availability of the code used to analyse the data (raw or simulated) or where the link to the code was no longer working

### Intervention period

We defined the intervention period based on the policy change date at *Biostatistics* and using the article’s submission date as this is when authors are thinking about the journal requirements and perhaps becoming aware of the badge. Since the policy change was on 1 July 2009, papers submitted to *Biostatistics* after that date were in the intervention period. We included a six month gap before the policy change as an interim phase because papers submitted during this time (1 January 2009 to 1 July 2009) could experience the badge policy upon re-submission, so papers submitted in this period were categorized into the interim period. Any papers submitted to *Biostatistics* before 1 January 2009 were in the control period and all papers submitted to *Statistics in Medicin*e were controls.

The first analysis examined data and code availability and probability of sharing over time using submission date. As a sensitivity analysis, we used the articles’ publication dates extracted from PubMed in place of submission date. We conducted this sensitivity analysis to examine whether the policy was associated with a change based on the very latest date that authors could make changes to their papers.

### Statistics methods

We plotted the binary data and code sharing over time and included a smooth curve to estimate the mean sharing rate over time in each journal. The smooth curves were made using a LOESS smooth with a span of 0.9, and we also plotted the 95% confidence intervals. Papers where there was no data to share (i.e., using simulated data) were excluded from these plots.

To test for a difference in the probability of making data and code available after the introduction of badges, we used logistic regression and presented the results as prevalence ratios rather than odds ratios, as prevalence ratios are generally easier to understand ^14^. Due to possible convergence issues with a standard logistic regression model using a log-link to estimate prevalence ratios, we ran a Bayesian logistic regression model using WinBUGS (version 1.3.4). Using a Bayesian model has the added advantage of giving 95% credible intervals and Bayesian p-values that are far easier to interpret than frequentist confidence intervals and p-values. The Bayesian p-values used here estimate the probability that sharing increased after the policy change at *Biostatistics*. As well as showing the change in data and code sharing probability, on the relative scale, of the prevalence ratio, we also show the absolute increase in sharing probability after the policy change together with 95% credible intervals.

In a sensitivity analysis we used a strong control for time by including year as a random effect, assuming that each year has its own data sharing rate. This essentially matches papers from *Biostatistics* and *Statistics in Medicine* from the same year. We did this to adjust for other changes over time, for example a potential increase over time in data and code depositories such as GitHub, Figshare, and Dryad, and a potential decrease in data and code availability for papers published many years ago because of broken links ^15^.

The current editors of *Biostatistics* indicated that when the publisher (Oxford) switched to a new publishing platform in January 2017, some of the supplemental material was lost in the transfer (personal communication, J Leek, 8 November 2017). As such, we conducted a sensitivity analysis assuming these broken links worked before Oxford changed publishing platforms.

The data analysis was made using the statistical software R (version 3.2.3).

## Results

### Broken links

We often encountered issues with broken hyperlinks at both journals. Forty-nine out of 76 (64%) articles that provided links to data and code at *Biostatistics* had broken links and at *Statistics in Medicine,* 21 out of 53 (40%) articles that provided links to data and code had broken links. We examine the impact of these broken links in sensitivity analyses.

### Data availability over time

**Figure 1.**
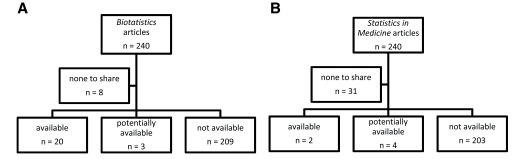
**a**: Flow chart of data availability. Randomly selected *Biostatistics* articles from 2006 to 2013, **b**: Flow charts of data availability. Randomly selected *Statistics in Medicine* articles from 2006 to 2013.

Flow charts show the frequency of data and code availability for each journal ( Figures 1a and 1b). *Biostatistics* had 8 articles with no data to share, bringing the sample with possible data available to 232; 20 of which had data available, 3 had data potentially available and 209 had no data available. *Statistics in Medicine* had 31 articles with no data to share, bringing the sample with possible data available to 209; 2 of which had data available, 4 had data potentially available and 203 had no data available.

**Figure 2.**
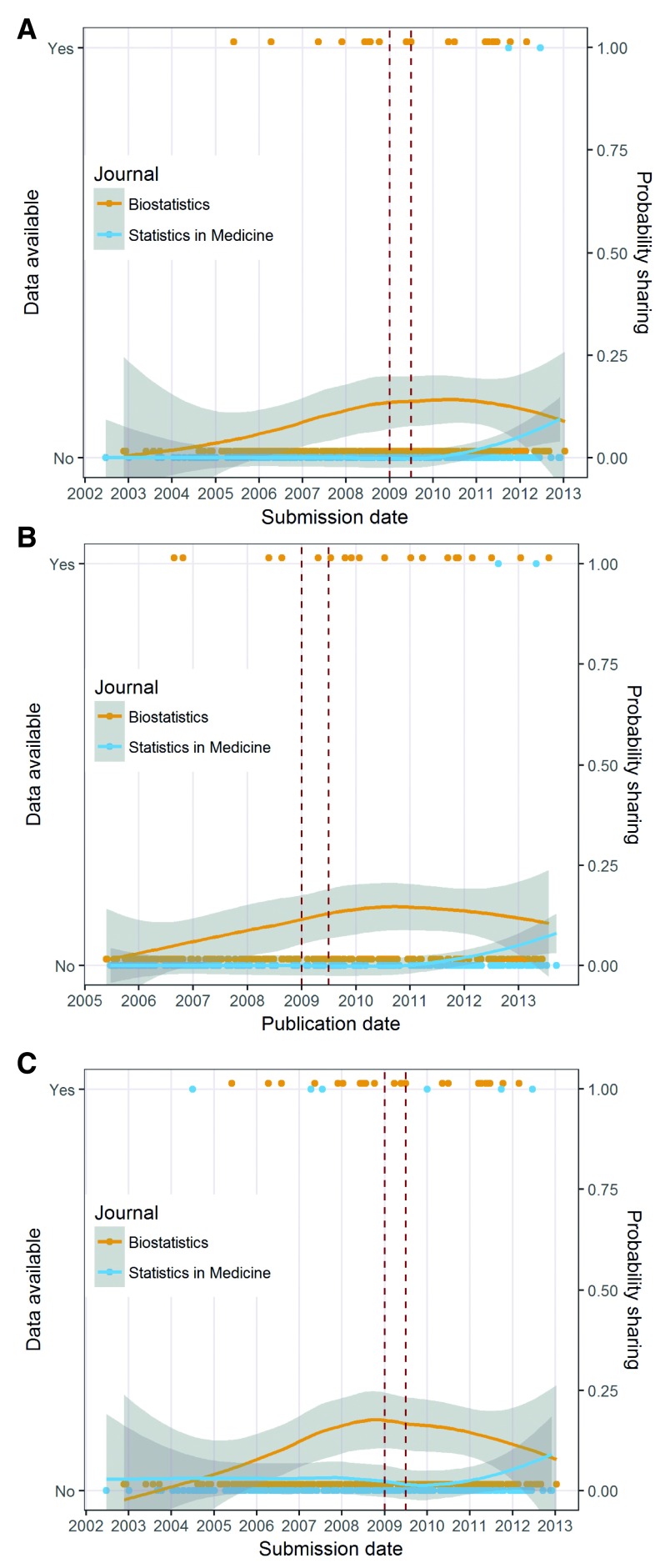
**a**: Plot of data availability over time by submission date. The dots at ‘No’ or ‘Yes’ are individual articles and the lines are a smoothed mean using a LOESS together with 95% confidence intervals (grey areas). The red lines indicate the interim period: 1 January 2009 to 1 July 2009. **b**: Plot of data availability over time by publication date. The dots at ‘No’ or ‘Yes’ are individual articles and the lines are a smoothed mean using a LOESS together with 95% confidence intervals (grey areas). The red lines indicate the interim period: 1 January 2009 to 1 July 2009. **c:** Plot of data availability by submission date assuming the now broken links were working at the time. The dots at ‘No’ or ‘Yes’ are individual articles and the lines are a smoothed mean using a LOESS together with 95% confidence intervals (grey areas). The red lines indicate the interim period: 1 January 2009 to 1 July 2009.

The data available and probability of sharing by submission date together with a smooth mean and 95% confidence intervals are in Figure 2a. The vertical red lines are at 1 July 2009, the date badges were introduced at *Biostatistics*, and 1 January 2009, six months prior to the policy change (interim period). It is clear that data availability and probability of sharing were greater over time in *Biostatistics* than in the control journal, *Statistics in Medicine*, but the probability of sharing data at *Biostatistics* was still low, at well below 0.25. Interestingly an increase in data sharing at *Biostatistics* took place before badges were introduced at the journal. The results of the sensitivity analysis using publication date are shown in Figure 2b. The smooth means in Figure 2b are similar to those in Figure 2a and show that data availability and probability of sharing were increasing at *Biostatistics* before badges were introduced. The results of the sensitivity analysis assuming the broken links were working using submission date as the time variable are shown in Figure 2c. The smooth means in Figure 2c are similar to those in Figures 2a and 2b, showing that the data sharing results are not greatly influenced by these broken links.

***Code availability over time***.

**Figure 3.**
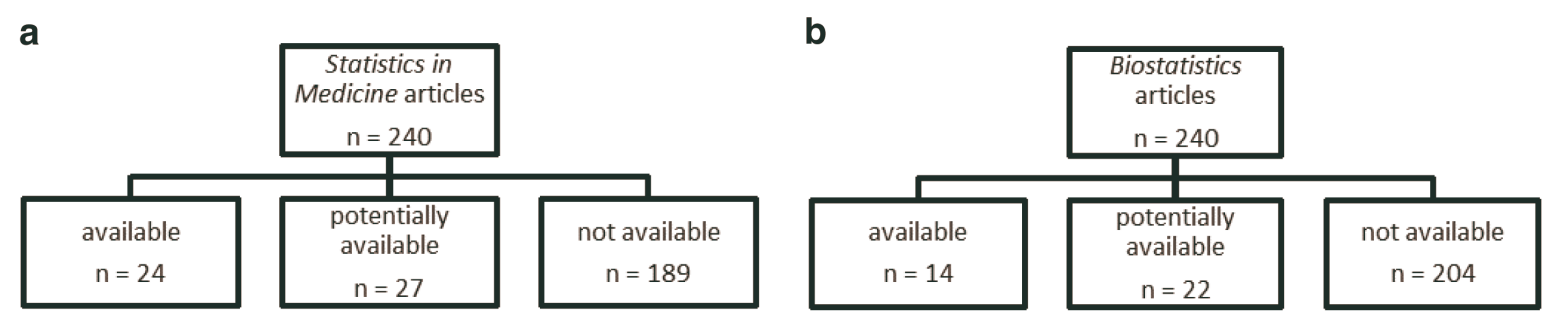
**a**: Flow charts of code availability. Randomly selected *Biostatistics* articles from 2006 to 2013, **b**: Flow charts of code availability. Randomly selected *Statistics in Medicine* articles from 2006 to 2013.

The frequency of code availability for each journal is in Figures 3a and 3b, which were comparable for the two journals. *Statistics in Medicine* had 24 articles with code available, 27 potentially available, and 189 with no code available, while *Biostatistics* had 14 articles with code available, 22 potentially available, and 204 with no code available.

**Figure 4.**
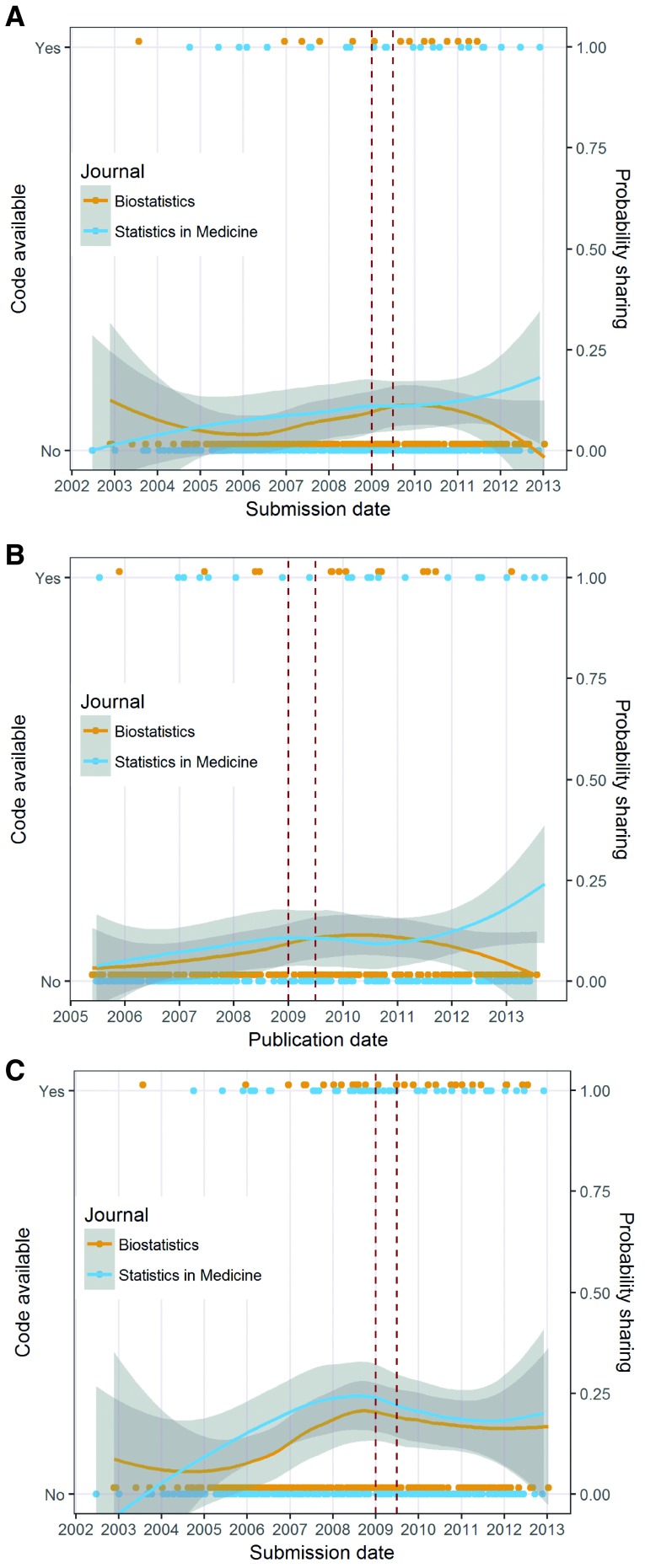
**a**: Plot of code availability over time by submission date. The dots at ‘No’ or ‘Yes’ are individual articles and the lines are a smoothed mean using a LOESS together with 95% confidence intervals (grey areas). The red lines indicate the interim period: 1 January 2009 to 1 July 2009. **b**: Plot of code availability over time by publication date. The dots at ‘No’ or ‘Yes’ are individual articles and the lines are a smoothed mean using a LOESS together with 95% confidence intervals (grey areas). The red lines indicate the interim period: 1 January 2009 to 1 July 2009. **c:** Plot of code availability by submission date assuming the now broken links were working at the time. The dots at ‘No’ or ‘Yes’ are individual articles and the lines are a smoothed mean using a LOESS together with 95% confidence intervals (grey areas). The red lines indicate the interim period: 1 January 2009 to 1 July 2009.

The code availability and probability of sharing by submission date together with a smooth curve and 95% confidence intervals are in Figure 4a. The smooth means for *Biostatistics* and *Statistics in Medicine* are mostly on top of each other in this graph, except for a drop-off in sharing at *Biostatistics* in later years. This indicates no great difference in code sharing at these journals. Figure 4b shows the results of the sensitivity analysis, where publication date was used instead of submission date. In this graph ( Figure 4b), the smooth curves for *Biostatistics* and *Statistics in Medicine* are again mostly on top of each other, showing an increase in code sharing over time at both journals, but around mid-2011 the two curves diverged, with *Statistics in Medicine* showing an increase in code sharing and *Biostatistics* a drop. The results of the sensitivity analysis assuming the broken links were working using submission date as the time variable are shown in Figure 4c. In this graph the smooth curves are again mostly overlapping, but with greater code availability over time at both journals.

### Increase in data sharing associated with badges

The logistic regression model estimated that the probability of data sharing increased by 5.7 (95% CI for prevalence ratio: 0.69 to 16.43, p-value: 0.947) times that of the control period in the interim period of 1 January 2009 to 1 July 2009. This Bayesian p-value gives an estimated 94.7% probability that the mean rate of sharing increased. After the interim period, the probability of data sharing increased by an estimated 3.9 (95% CI: 1.5 to 8.4, p-value: 0.998) times after badges were introduced. On an absolute scale, this difference was only a 7.6% increase in data sharing (95% CI: 2 to 15%). After controlling for time, badges increased the probability of data sharing at the journal by an estimated 4.9 times (95% CI: 1.5 to 13.0, p-value: 0.997). This is comparable to the prevalence ratio of 3.9 when time was not added as a random effect, which shows that controlling for time only slightly increased the effect badges had on the probability of data sharing. After assuming the now broken links were working at the time of publication, the logistic regression model that controlled for time gave a slightly different estimate of the mean effect of badges from the previous 7.6% to 6.7% (95% CI: 0.0% to 17.0%, p-value: 0.974).

### Increase in code sharing associated with badges

During the interim period, badges did not have an effect on code sharing (prevalence ratio of 1). After the interim period there was an estimated 0.61% increase (95% CI: –5 to 8%, p-value: 0.55) in sharing. After adjusting for time, this absolute difference reduced to –1.4% (95% CI: –7 to 5%, p-value: 0.287). This suggests that badges did not have an impact on the probability of sharing code. After assuming the now broken links were working at the time of publication, the logistic regression model that controlled for time estimate a slightly changed mean effect of badges from the previous 0.61% to –2% (95% CI: –10 to 7%, p-value: 0.286).

## Discussion

### Are badges effective incentives for reproducibility?

The results of this observational study and those of the related Kidwell *et al*. badge study ^8^ cannot accurately deduce the effectiveness of badges because of the biases of the non-randomised study design. The Kidwell *et al*. 2016 badge study received criticism from Hilda Bastian on its study design, analyses, and claims ^16^. One of the criticisms was that the badges scheme was not the only intervention offered at the journal, there were four other co-interventions offered in 2014, and so any effect could not be attributed to badges alone ^16^. Bastian reasonably argued that to isolate the impact of badges, groups that had the same conditions except badges were needed ^16^. Our study is also exposed to similar limitations with regard to confounding as other changes may have occurred that we were not aware of. However, we can derive some insight into the effect badges had on data and code sharing from the results of both observational studies.

After the introduction of badges at *Biostatistics,* the probability of data sharing increased 3.9 times. This prevalence ratio might seem like a large increase but on an absolute scale it is only a 7.6% increase in the rate of data sharing, which is much lower than the 37.9% effect of badges at *Psychological Science* ^8^. When the now broken links were assumed to indicate sharing, the badge effect reduced slightly to 6.7%. The large difference between the effect of badges at *Biostatistics* and *Psychological Science* could be related to differences in the culture of sharing between the two fields, and the timeframes of the studies: 2006 to 2013 for our study, versus 2012 to 2015 for Kidwell *et al*. Our study analysed incentives for data and code sharing at an earlier time when the reproducibility crisis was not yet a testified reality, hence researchers may have been more primed to change behaviour in the Kidwell *et al*. study. Also, since statisticians typically re-analyse existing datasets, it might be harder for them to share the data as they might not have the rights. This is contrary to research in psychological science where original data is normally collected and analysed, making sharing a potentially simpler task.

There was an apparent increase in data sharing before badges were introduced at *Biostatistics* ( Figure 2a). One possibility is that articles that were submitted before the policy change could still have experienced the policy because of the time needed for peer review and resubmission. We used submission date to determine if articles were prepared before or after the policy change because we know that sharing data often takes preparation time and we believed that authors were therefore more likely to react to the policy when they were writing their first draft. However, data sharing seemed to be increasing before badges were introduced even when we used publication date in a sensitivity analysis. The reproducibility policy at *Biostatistics* was built on the existing framework that “allowed and encouraged authors to place supplementary materials online” ^1^. Such an option of depositing supplementary material could have contributed to the rise in data sharing before badges. Also, Roger Peng assumed the role as the Associate Editor for reproducibility at *Biostatistics* in 2006, which might have catalysed a change in the culture of reproducibility at the journal. Another possible contributor to the increase in data sharing before the policy change is the general trend towards more open science and open data ^17^.

Badges did not appear to have an effect on code sharing as the prevalence ratio was 1.1 When the now broken links were assumed to indicate code sharing, the badge effect on code changed slightly from 0.61% to –2%. This is an unexpected outcome as code is of great importance in the field of biostatistics. A possible explanation behind the lack of badge effect on code sharing could be our definition of code sharing, which might seem traditional compared with the reproducibility policy at *Biostatistics*. We defined code sharing as the availability of the code used to analyse the data (original or simulated) in the article. The policy at *Biostatistics* included referencing “…software that is widely available from central repositories (e.g. CRAN, Statlib)”. It is true that providing a link to a third-party repository where software packages are deposited, such as vignettes, typically contain some general code, but it often takes specialized skills to work out the code at these repositories, and they might not always explain the analyses covered in the actual published article. This is in line with what Stodden *et al*. recommended in their piece on reproducibility in *Science*, “Data and code underlying discoveries must be discoverable from the related publication, accessible, and reuseable” ^18^.

Badges have been promoted as a simple solution because they are low cost. However, while collecting data for our study, we noticed that articles did not always appear to be allocated with badges correctly, implying that assigning badges is not always clear cut and journal staff may need to spend more time on verification. An alternative approach is that peer-reviewers check for data and code availability and assign badges as part of the standard peer review process. It could be that peer-reviewers prefer to have access to the data and code in order to review the article anyway, so this model might work, but it still requires additional time and effort on their part and as they receive little recognition for their work, plus it might be unfair to expect all peer-reviewers to check for data and code sharing.

## Conclusion

Efforts are underway by the global meta-research community to strengthen the reliability of the scientific method ^19^. Data and code sharing is an indispensable part of the movement towards science that is open; where scientific truth is not a questionable commodity, but is easily accessible, replicable, and verifiable ^20^. The cultural shift towards reproducible science is complex and it calls for a twofold change in the attitudes of individual researchers toward reproducibility, and the leadership provided by the systems and services that support scientific research. As such, journals, universities, government bodies, and funders are key players in promoting this culture. Transparency and reproducibility are elements central to strengthening the scientific method, and data and code provide the key to scientific truth ^12^. As Peng argued in *Science*, the culture of reproducibility will not drastically change overnight, but simply bringing the notion of reproducibility to the fore and making it routine will make a difference ^3^. Badges are already being used by journals including *Biostatistics*, *Psychological Science*, *British Medical Journal Open Science*, and *Association for Computing Machinery* to encourage researchers to share the evidence behind their work ^1, 21^. Based on this observational study and a previous study, it appears that badges do help to increase data sharing, but a randomised trial is needed to better estimate their true effect, as well as studies of the additional time needed to implement and maintain them.

## Data availability

Anonymised data and the R code used in the analyses are publicly available at: https://doi.org/10.6084/m9.figshare.5687548.v2 ^22^

## Consent

An ethics exemption was granted by the Office of Research Ethics and Integrity at the Queensland University of Technology for this study (exemption number: 1700001051). No consent was needed as all data collected and analysed in this study were publicly available.
