# Supplementary material for: Badges for sharing data and code at Biostatistics: an observational study
Source: F1000Res. 2018 Mar 7;7:90. Originally published 2018 Jan 19. [Version 2] doi: 10.12688/f1000research.13477.2 (PMC5843843; doi:10.12688/f1000research.13477.2)
Supplement: Peer review history

# Peer review: Referee response for version 2

**DOI:** 10.5256/f1000research.15304.r31606

**Published:** 2018-03-08

**Authors:** Bastian Greshake

**Affiliations:** 1 Division of Environmental Genomics and Systems Biology, Lawrence Berkeley National Laboratory, Berkeley, CA, USA; 2 Open Humans (openhumans.org), Frankfurt am Main, Germany

The authors did a great job and addressed the comments to the first version of this manuscript in good way. Thus I fully approve of this version!

I have read this submission. I believe that I have an appropriate level of expertise to confirm that it is of an acceptable scientific standard.

# Peer review: Referee response for version 2

**DOI:** 10.5256/f1000research.15304.r31607

**Published:** 2018-03-07

**Authors:** Gustav Nilsonne

**Affiliations:** 1 Stress Research Institute, Stockholm University, Stockholm, Sweden; 2 Department of Clinical Neuroscience, Karolinska Institutet, Stockholm, Sweden

The revision addresses the issues raised in the first round of review. The rationale for inclusion is now clearer, and the sensitivity analyses are helpful. I am happy to approve this article again.

I have read this submission. I believe that I have an appropriate level of expertise to confirm that it is of an acceptable scientific standard.

# Peer review: Referee response for version 1

**DOI:** 10.5256/f1000research.14635.r30239

**Published:** 2018-02-12

**Authors:** Bastian Greshake

**Affiliations:** 1 Division of Environmental Genomics and Systems Biology, Lawrence Berkeley National Laboratory, Berkeley, CA, USA; 2 Open Humans (openhumans.org), Frankfurt am Main, Germany

This study evaluates the effects that badging has on availability of both data and source code. Badges that highlight and reward articles that share data/code have been described as a simple way to increase sharing behavior and thus reproducibility. This makes this study highly relevant and interesting to the field of meta-research. To measure whether badging has this desired effect the authors compare the availability of data/code in the journal *Biostatistics* - which introduced badges in 2009 - with a control journal, *Statistics in Medicine. *The main finding is that there seems to be a small effect on data sharing associated with the introduction, while badges appear to have no effect on the sharing of code. 

 While the statistical methods are appropriate and sound, the study has some limitations given by the data set on how the authors coped with missing data. First of all, and this is probably the main limitation, there is only a small set of publications in the authors' set of 480 that have data/code associated with them. Only around 8% of all publications have code attached, a number that drops to less than 5% for data. Given this overall low rate of sharing - and the large time span covered - there is a lot of fluctuation in the observed sharing behavior for both code and data (c.f. Figure 3 & 4) between the two journals. Given that only *Statistics in Medicine *is used as a control journal it is unclear how much of these differences are cultural between the audiences of the two journals (also c.f. that code sharing is more prevalent in *SIM* than *Biostatistics *and increases more despite the lack of badges). A larger set of control-journals would potentially minimize this 'culture'-effect (though requiring a potentially unreasonably large time investment).

 In addition to this there are further effects that complicate a clear inference of the effect of badges:

1. There is a general trend that "Open Science" and data sharing are becoming more popular (c.f. Tenopir et al. (2015) ^1^). In line with this the authors find that the sharing of data in *Biostatistics* already increased prior to the introduction of badges, even when going for conservative measures. Additionally there is an observed increase in data sharing in *Statistics in Medicine* in later years, albeit from a lower baseline. I think this general trend should be included in the discussion.
2. The authors additionally find that the links for 64% of articles that provide data/code are broken in *Biostatistics* and highlight the issue that OUP had with losing (not only) supplementary data. The authors treat these articles as having data/code not publicly available. This not only leads to a marked decrease overall decrease of articles with data/code available but can furthermore be a potential source of bias. For OUPs *Molecular Biology and Evolution* it was seen that these losses on behalf of OUP not only affected supplementary materials but even individual years worth of full text articles (c.f. http://rossmounce.co.uk/2017/04/02/open-letter-to-oxford-university-press/). If OUP also lost the supplementary materials of certain publication date ranges for *Biostatistics* this will heavily bias the analysis presented here. 

Given these limitations I would be cautious to assume whether badges have a positive effect or not on data availability at this point.

 To improve the resolution by adding more data and to decrease the biases mentioned in *2)* I recommend taking the original data/code availability statements at face value and not treat broken links as publications without data/code. I think it is defendable to assume that the data/code was available at the time of publication when the main text suggests so. Doing this the potential effect of badges should become more pronounced as it is not hidden by the 'link-rot' that OUP is responsible for.  

 Overall, I think this is a valuable contribution to the field of meta-research and our understanding of incentives around sharing and reproducibility. I hope that the minor improvements suggested above can be useful to make this data set even more useful.

I have read this submission. I believe that I have an appropriate level of expertise to confirm that it is of an acceptable scientific standard, however I have significant reservations, as outlined above.

## References

[1] TenopirCDaltonEAllardSFrameMPjesivacIBirchBPollockDDorsettK: Changes in Data Sharing and Data Reuse Practices and Perceptions among Scientists Worldwide. .2015;10(8) : 10.1371/journal.pone.0134826 10.1371/journal.pone.0134826 PMC455024626308551

# Response

**Published:** 2018-03-01

**Authors:** Anisa Rowhani-Farid

**Affiliations:** Queensland University of Technology, Australia

We looked for additional control journals to minimise the ‘culture’-effect described above.  The journals that met the eligibility criteria of being in the field of applied statistics and/or biostatistics and which had a similar relative high prestige in the field as per *Biostatistics *were the following:

*Statistical Methods in Medical Research*

*Biometric Journal*

*Biometrics*

*Royal Statistical Society – Series C Applied Statistics * 

 However, none of these journals were suitable as controls as they all had reproducibility policy changes during the time frame of our study (2006 to 2013) or had a reproducibility policy in place before that timeframe.  We have added an explanation of this in our methods.    

 The general trend towards more open science is now discussed in the Discussion. We used sensitivity analyses that adjusted for the effects of time (including trends), and the effects of badges were relatively similar to the unadjusted results.

 We have conducted sensitivity analyses for data and code sharing to account for the broken links at the journal. These results have been added and discussed in the article.

# Peer review: Referee response for version 1

**DOI:** 10.5256/f1000research.14635.r30171

**Published:** 2018-01-24

**Authors:** Gustav Nilsonne

**Affiliations:** 1 Stress Research Institute, Stockholm University, Stockholm, Sweden; 2 Department of Clinical Neuroscience, Karolinska Institutet, Stockholm, Sweden

This study is an observational investigation of the effect of badges/kitemarks on sharing of data and code. The authors compared one journal that introduced badges in 2009 ( *Biostatistics*) to one that did not ( *Statistics in Medicine*). The main finding was that badges were associated with an increase in open data publication, although with a considerably smaller effect than that observed in the one previous investigation of this question. The finding is important and may influence publishing policies.

 The main limitation of this study is that only two journals were included. The authors provide a strong reason for this, namely that *Biostatistics *is unique in having introduced badges sufficiently long ago to have a period of follow-up of several years.

 Statistical methods are sound; descriptive statistics are very clear and inferential statistics are appropriate. The choice to use the six months before badges came into effect (the "interim period") as a reference period is arbitrary, and it is not possible for a reader to assess whether six months would capture most papers undergoing review and revision at the time the policy was introduced. If papers took longer, that could contribute to the increasing rates of data sharing observed in the run-up to the introduction of badges, seen in figure 3. Thus, the choice of reference period is likely to yield a conservative estimate of the effect of the policy.

 Papers were coded by one person only, and that is a minor weakness. My experience with coding articles for open data and similar outcomes leads me to think that the main reason to have two coders in this context is to reduce the risk of error, rather than to handle interrater differences in assessments. It is not likely, in my opinion, that errors in the coding have effects on a magnitude approaching that seen for the intervention.

 I have cursorily reviewed the data and analysis code also. The data variable names are self-explanatory. The code, written in Markdown, is sufficiently documented with inline headings. However, the code requires some modification before it can be run independently, and this is not documented. For example, on line 18, the code attempts to load the data, by calling a file that is not provided. The data must instead be loaded by opening the provided R workspace file. I suggest that the data be made available in a csv or txt file, which is safer for accessibility in the long term and across softwares. The code could then call this file.

 The ancillary finding of high rates of broken hyperlinks to data at both journals is interesting, as is the explanation that supplementary data were lost by one of the journals during a platform migration. I have several times advanced the argument that this risk is one motivation for publishing data and code using dedicated repositories, but I have not previously seen an empirical observation such as this one. I suggest that this finding should be mentioned in the abstract.

 One minor detail: In figure 4, the legend does not explain the difference between panels a and b, as in figure 3.

 In summary, this article makes an important contribution towards our understanding of effects of badges on publication of open data and open code. I am happy to approve this article.

I have read this submission. I believe that I have an appropriate level of expertise to confirm that it is of an acceptable scientific standard.

# Response

**Published:** 2018-03-01

**Authors:** Anisa Rowhani-Farid

**Affiliations:** Queensland University of Technology, Australia

We have updated the Rmarkdown code and added an R data set so anyone can independently run the analysis.

 Although ARF coded all the data, AGB verified 20 observations and the two authors debated a handful of cases where it was not clear. In general the sharing of data or not was relatively clear-cut and hence we believe that one coder should be sufficient.

 We agree that the six month date window prior to the introduction of badges is arbitrary, and this is why we also plotted the sharing rates over time so that readers could see when any changes in sharing took place, and why we tried the alternative time scale of submission date.

 We have now mentioned broken links in in the abstract.

 We have corrected this: "One minor detail: In figure 4, the legend does not explain the difference between panels a and b, as in figure 3". 
